# Novel variations in the PLOD1, COL1A1, COL5A2 and COL4A1 genes related to keratoconus

**DOI:** 10.3389/fgene.2025.1497915

**Published:** 2025-03-25

**Authors:** Qinghong Lin, Xuejun Wang, Xiaoliao Peng, Tian Han, Ling Sun, Xiaoyu Zhang, Xingtao Zhou

**Affiliations:** ^1^ Department of Ophthalmology, Eye and ENT Hospital of Fudan University, Shanghai, China; ^2^ Eye Institute and Department of Ophthalmology, Eye and ENT Hospital, Fudan University, Shanghai, China; ^3^ NHC Key Laboratory of Myopia (Fudan University), Key Laboratory of Myopia, Chinese Academy of Medical Sciences, Shanghai, China; ^4^ Refractive Surgery Department, Bright Eye Hospital, Fuzhou, China

**Keywords:** keratoconus, procollagen-lysine, 2-oxoglutarate 5-dioxygenase 1 (PLOD1), collagen type V alpha 2, collagen type I alpha 1, collagen type IV alpha 1, early diagnosis

## Abstract

**Purpose:**

To investigate the genetic characteristics of four Chinese families affected by keratoconus (KC).

**Methods:**

In the four families affected by KC, medical records, clinical observations, and blood samples were collected from all individuals. One hundred subjects without KC served as healthy controls. All controls and subjects in the four families underwent whole exome sequencing of their genomic DNA and polymerase chain reaction to confirm the variants. All variants were analyzed using online software; and *in silico* predictions of three-dimensional protein structures were performed.

**Results:**

The clinical manifestations in those first-degree family members of the probands were atypical. The following four variants were identified in the four probands and other family members with KC: heterozygous missense variation c.109G>A (p.Glu37Lys, rs369263247) in the procollagen-lysine, 2-oxoglutarate 5-dioxygenase 1 (*PLOD1*) gene; heterozygous missense variation c.3766G>A (p.Ala1256Thr, rs148216434) in the collagen type I alpha 1 (*COL1A1*) gene; heterozygous missense variant c.4364G>A (p.Gly1455Glu) in the collagen type V alpha 2 (*COL5A2*) gene; and missense variation c.976G>A (p.Glu326Ser) in the collagen type IV alpha 1 (*COL4A1*) gene. The above genotypes were co-segregated with corresponding phenotypes. All variations in these families appeared to be pathogenic.

**Conclusion:**

Four variants in the *PLOD1*, *COL1A1*, *COL5A2*, and *COL4A1* genes were identified in this study, which are collagen-coding genes and collagen crosslink regulatory genes and may be associated with the origin and development of KC. This study updates the knowledge of genes related to KC and the biomedical implications.

## Introduction

Keratoconus (KC) is a corneal disorder characterized by thinning of the cornea, conical protrusion, irregular astigmatism, and impaired vision, typically emerging during adolescence (Rabinowitz, 1998). KC is genetically characterized by a complex polygenic inheritance involving multiple susceptibility loci and environmental interactions, such as eye rubbing. About 5%–14% of KC patients have a family history (Bykhovskaya et al., 2016; Loukovitis et al., 2018). Collagens are the major component of the cornea stroma. The interaction between collagens and proteoglycans maintains the normal optical function of the cornea (Egrilmez et al., 2004; Legare et al., 2013). However, in the cornea of a patient with KC, there is decreased total collagen and the crosslinking of collagen molecules is defective (Farjadnia and Naderan, 2015). This can cause the cornea to thin and protrude forward due to reduced mechanical resistance. Therapies such as ultraviolet-A (UVA) crosslinking with riboflavin treat KC by increasing the mechanical resistance of the cornea involving anti-collagenase activity (Wu et al., 2021).

In the last two decades, accompanied by the advancement and prevalence of myopia correction surgery, young patients with myopia come in great numbers to the clinic. Through comprehensive pre-operative ocular examinations, an increasing number of corneal morphological changes have been identified and prompted further exploration of KC, especially the incipient stage of the disease. Therefore, identifying genetic susceptibility to KC is very important in the early diagnosis of these myopia correction surgery candidates. Numerous genes code for various collagens in the cornea stroma; their association with the central cornea thickness (CCT) and KC have been studied such as *COL1A1*, *COLIA2*, *COL4A3*, *COL4A4*, *COL5A1*, and *COL8A2* (Dimasi et al., 2010; Vithana et al., 2011; Gao et al., 2013; Li et al., 2013; Abu-Amero et al., 2015; Saravani et al., 2015; Hosoda et al., 2020). Thus, further exploring genetic alterations in collagen-related genes is meaningful and will assist in the early detection and management of KC.

In the present study, we recruited four Chinese families with KC to evaluate their genetic characteristics. Four variants were identified and analyzed, and their biomedical implications are discussed.

## Materials and methods

### Participants and examinations

This study enrolled 124 people including 24 family members from four Chinese families with KC and 100 healthy Chinese individuals without KC. Among the 24 family members, 4 were from family 1 (2 patients and 2 healthy members), 7 from family 2 (4 patients and 3 healthy members), 10 from family 3 (5 patients and 5 healthy members), and 3 from family 4 (2 patients and 1 healthy member). The 100 unrelated healthy individuals served as controls; they were diagnosed with refractive errors but without KC or any other corneal disease.

This study adhered to the Chinese Expert Consensus on the Diagnosis and Treatment of Keratoconus (2019) for the clinical diagnostic criteria and stages of KC (Chinese Medical Association Ophthalmology Branch Corneal Disease Group, 2019; Shi et al., 2019). Written informed consent was provided by all subjects. All participants received an examination, which included VA, anterior and posterior segments of the eye, and corneal evaluation using the Scheimpflug Camera System (Pentacam; Oculus Optikgeräte GmbH, Wetzlar, Germany). Approval for this study was obtained from the institutional review board of Fudan University (Shanghai, China) (Approval No. 2022128), and all procedures were carried out in accordance with the principles of the Declaration of Helsinki.

### Whole exome sequencing

Exome sequencing (ES) was performed for 15 participants (all 4 members of family 1; Ⅲ1, Ⅲ2, Ⅱ3, and Ⅱ4 in family 2; Ⅳ1, Ⅲ1, Ⅲ2, and Ⅲ3 in family 3; all 3 members of family 4) using the method described in our previous study (Lin et al., 2023). Leukocyte-derived DNA was extracted, followed by ES and enrichment of exonic sequences from the genomic DNA of KC subjects. Subsequently, the loci that met the criteria for co-segregation in the four pedigrees were listed in the Supplementary Table S1. Considering population frequencies, genes related to the cornea, functional mutations in exonic regions or splice site regions, and conducting first-generation sequencing verification for all members of the pedigrees at the listed loci in Supplementary Table S1, the loci identified were ultimately selected as suspected mutations causing the diseases in the mentioned pedigrees. Reported variants (e.g., transforming growth factor beta-induced) and those occurring in corneal disorder, especially KC subjects at frequencies ≤1%, were assessed using the 1000 Genomes Project. Only variants co-segregating with KC in affected family members were considered candidate variants.

### Variant validation and cross-species conservation analyses

Variant validation and analyses were conducted following the methodology outlined in our previous work (Lin et al., 2023). All variants underwent analysis using online software tools as detailed in our published report (Lin et al., 2023) including Polyphen2, Sorting Intolerant from Tolerant, Protein Variation Effect Analyzer, fathmmMKL, and Mutation Taster. In addition, CADD v1.4 (http://cadd.gs.washington.edu) and VarSite datebase (www.ebi.ac.uk), with scoring in accordance with the American College of Medical Genetics and Genomics (ACMG) guidelines (Richards et al., 2015). Genomic Evolutionary Rate Profiling ++ (GERP++) (https://bio.tools/gerp) was used to predict the conservation of the variants.

To validate the candidate variants, polymerase chain reaction (PCR) and Sanger sequencing were performed on the other family members in the study and healthy controls. Primer3 was utilized for PCR primer design. The NCBI VARIANT, NCBI HomoloGene, and 1000 Genomes Project databases were used for validation and analysis. Three-dimensional (3D) protein structures of the variants were generated utilizing the online server Iterative Threading ASSEmbly Refinement (I-TASSER; https://zhanggroup.org/I-TASSER/).

### Analyses of the protein-protein interaction network

Analyses of the protein–protein interaction (PPI) network were performed using the online Search Tool for the Retrieval Interacting Genes 11.5 (STRING) (https://cn.string-db.org). Then Cytoscape (v3.9.0) was applied to study the network of interactions. The filter of crucial proteins was set at a degree ≥5.

## Results

### Clinical symptoms

**FIGURE 1:**
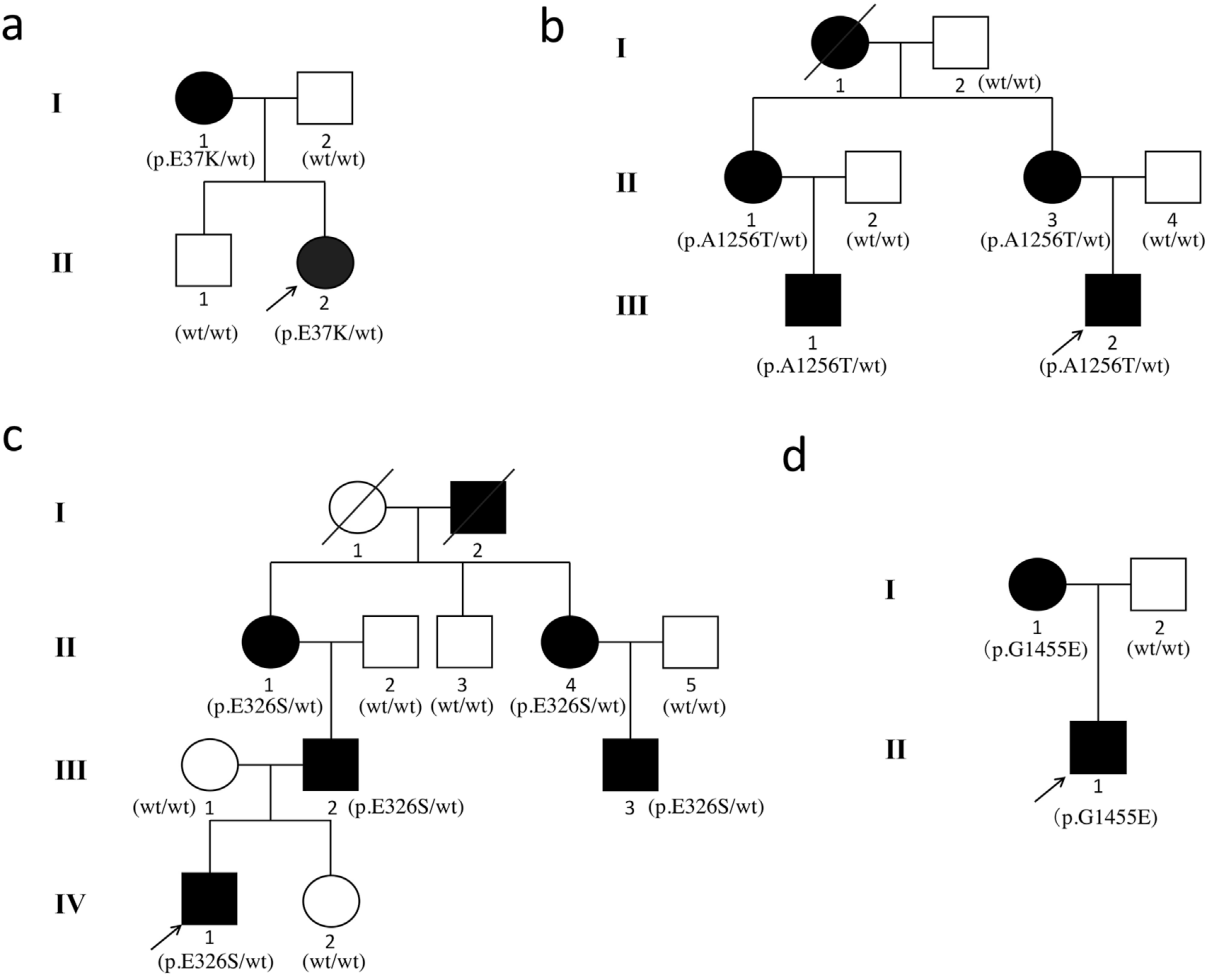
Pedigrees of the Chinese families with keratoconus. **(a)** family 1; **(b)** family 2; **(c)** family 3; and **(d)** family 4. The squares represent the males, and the circles represent the females. The open symbols indicate the unaffected family members. The solid symbols indicate the infected individuals. The diagonal line indicates the family member who passed away. The arrows indicate the probands.

**FIGURE 2:**
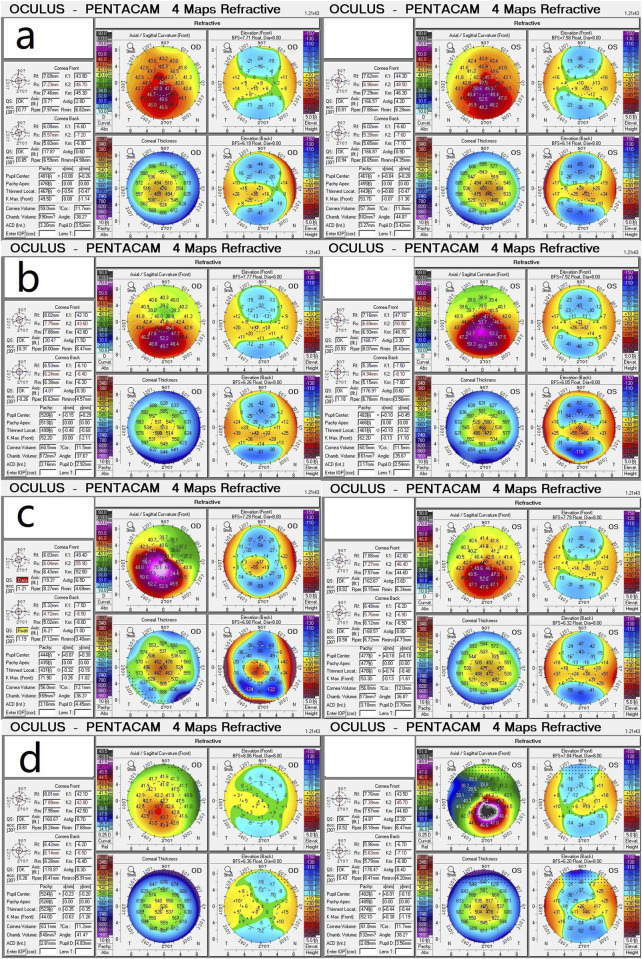
Corneal topography (Pentacam) reports show the mean values of posterior elevation of the cornea, maximum anterior surface curvatures, and central corneal thicknesses. **(a)** Proband (II.2) in family 1; **(b)** Proband (III.2) in family 2; **(c)** Proband (Ⅳ1) in family 3; **(d)** Proband (II.1) in family 4.

Figure 1 displays the genograms (1a: family 1; 1b: family 2; 1c: family 3; and 1d: family 4) and shows autosomal dominant inheritance of KC in all families. Two subjects in family 1 were diagnosed with KC. An 18-year-old female in this family was the proband (II.2); she had impaired vision in both eyes and was diagnosed as KC grade 1 in the right eye and grade 2 in the left eye; another subject in family 1 was 41 years old and had latent stage KC in her right eye and grade 1 in her left eye. In family 2, a 22-year-old young man was the proband (III.2) and his right eye was KC grade 1 and left eye was KC grade 2; the second subject in family 2 was a 23-year-old male with KC grade 2 in his right eye and incipient stage in his left eye; the third one was a 43-year-old female who had the same diagnosis as the second subject; the fourth subject in family 2 was a 45-year-old female with KC grade 2 in her right eye and grade 1 in her left eye. The proband in family 3 was a 17-year-old male (Ⅳ1) with KC grade 2 in both eyes; the second subject in family 3 was a 40-year-old male with KC grade 1 in his right eye and grade 2 in his left eye; the third subject in family 3 was a 22-year-old male with KC incipient stage in the right eye and latent stage in the left eye; the fourth subject was a 59-year-old female with KC grade 2 in both eyes; the fifth subject in family 3 was a 55-year-old female with KC incipient stage in her right eye and grade 1 in her left eye. In family 4, the proband was a 23-year-old male (II.1) with KC latent stage in his right eye and grade 1 in his left eye; another subject was a 45-year-old female with KC grade 2 in her right eye and incipient stage in her left eye. Corneal topography (Pentacam) reports of these probands are present in Figure 2 to show the mean values of posterior elevation of the cornea (PEC), maximum anterior surface curvatures (MASCs), and CCTs. The clinical data and grades of all KC patients in these four families are summarized in Table 1.

**TABLE 1:** Clinical data of the KC patients.

KC member	Sex	Age at diagnosis	CCT (µm)	Kmax	PEC (µm)	Clinical grade
Family 1						
II.2	F	18	467 µm (OD), 443 µm (OS)	49.5D (OD), 53.7D (OS)	38 µm (OD), 53 µm (OS)	1 (OD), 2 (OS)
I.1	F	41	502 µm (OD), 483 µm (OS)	43.9D (OD), 49.3D (OS)	7 µm (OD), 18 µm (OS)	Latent stage (OD), 1 (OS)
Family 2						
III.2	M	22	499 µm (OD), 461 µm (OS)	52.2D (OD), 62.2D (OS)	42 µm (OD), 66 µm (OS)	1 (OD), 2 (OS)
III.1	M	23	418 µm (OD), 496 µm (OS)	70.6D (OD), 46.6D (OS)	72 µm (OD), 18 µm (OS)	2 (OD), incipient stage (OS)
II.3	F	43	406 µm (OD), 513 µm (OS)	71.5D (OD), 42.6D (OS)	123 µm (OD), 14 µm (OS)	2 (OD), incipient stage (OS)
II.1	F	45	435 µm (OD), 495 µm (OS)	73.1D (OD), 46.1D (OS)	81 µm (OD), 20 µm (OS)	2 (OD), 1 (OS)
Family 3						
Ⅳ1	M	17	431 µm (OD), 470 µm (OS)	71.9D (OD), 53.3D (OS)	85 µm (OD), 34 µm (OS)	2 (OD), 2 (OS)
III.2	M	40	472 µm (OD), 453 µm (OS)	52.1D (OD), 54.6D (OS)	46 µm (OD), 65 µm (OS)	1 (OD), 2 (OS)
III.3	M	22	487 µm (OD), 478 µm (OS)	44.8D (OD), 45.5D (OS)	14 µm (OD), 9 µm (OS)	incipient stage (OD), Latent stage (OS)
II.1	F	59	454 µm (OD), 468 µm (OS)	61.5D (OD), 54.0D (OS)	55 µm (OD), 39 µm (OS)	2 (OD), 2 (OS)
II.4	F	55	531 µm (OD), 508 µm (OS)	45.8D (OD), 49.5D (OS)	17 µm (OD), 33 µm (OS)	incipient stage (OD), 1 (OS)
Family 4						
II.1	M	23	523 µm (OD), 474 µm (OS)	44.0D (OD), 52.1D (OS)	8 µm (OD), 46 µm (OS)	Latent stage (OD), 1 (OS)
I.1	F	45	445 µm (OD), 474 µm (OS)	55.6D (OD), 48.0D (OS)	34 µm (OD), 13 µm (OS)	2 (OD), incipient stage (OS)

KC, keratoconus; CCT, central corneal thickness; PEC, posterior elevation of the cornea; D, diopter; OD, right eye; OS, left eye.

### Identification and analyses of the novel variants

**FIGURE 3:**
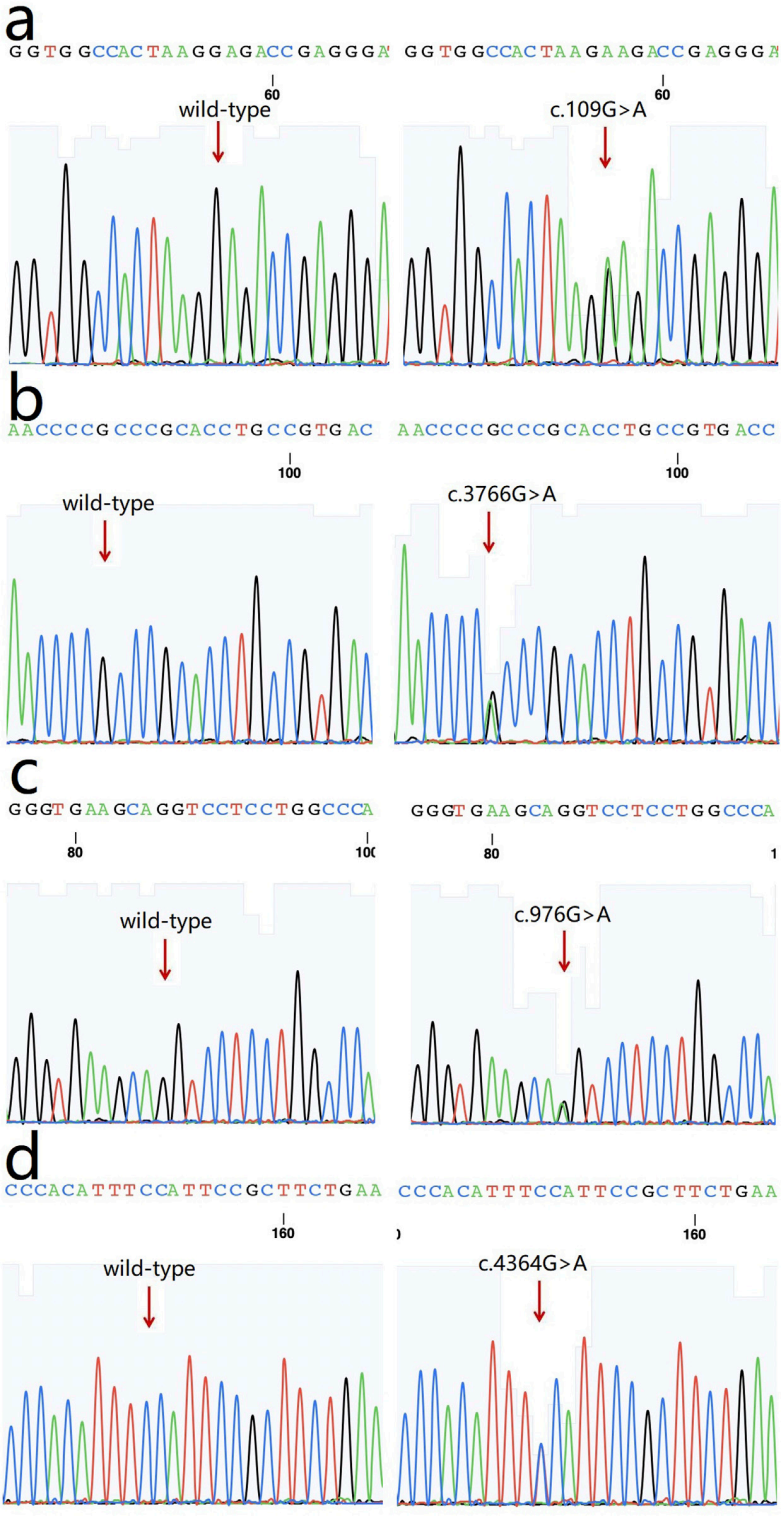
Sequence chromatograms of the variants. **(a)** c.109G>A (p.Glu37Lys, rs369263247) in *PLOD1* gene (arrow); **(b)** a heterozygous missense variation c.3766G>A (p.Ala1256Thr, rs148216434) in *COL1A1* gene (arrow); **(c)** a missense variation c.976G>A (p.Glu326Ser) in *COL4A1* gene; **(d)** a heterozygous missense variant c.4364G>A (p.Gly1455Glu) in *COL5A2* gene.

The following four variants were detected in the four families and were absent in the healthy controls: 1) a heterozygous missense variation c.109G>A (p.Glu37Lys, rs369263247) in the exon 2 of the procollagen-lysine, 2-oxoglutarate 5-dioxygenase 1 (PLOD1) gene in family 1 (Figure 3A). According to the VarSite database, a transition from a Glu to a Lys side chain is quite substantial, and could likely lead to a modification in the protein’s function. The variant residue is a lysine, possessing a positively charged side chain, thus enhancing its hydrophilicity. The amino acid alteration from a Glu to a Lys has a high “disease propensity” value of 1.14, and its occurrence in the gnomAD population database is exceedingly rare (PM2); 2) a heterozygous missense variation c.3766G>A(p.Ala1256Thr, rs148216434) in the exon 18 of the collagen type I alpha 1 (COL1A1) gene in family 2 (Figure 3B). Following the ACMG guidelines, its occurrence in the gnomAD population database is exceptionally rare (PM2) and is likely to have a deleterious effect (PP3); 3) a missense variation c.976G>A (p.Glu326Ser) in the collagen type IV alpha 1 (COL4A1) gene in family 3 (Figure 3C). Its occurence in the gnomAD population database is exceedingly rare (PM2), and it is likely to have a deleterious effect (PP3). In the VarSite database, residue alteration from a Gly to a Ser has a high ‘disease propensity’ value of 1.34; and 4) a heterozygous missense variant c.4364G>A (p.Gly1455Glu) in exon 54 of the collagen type V alpha 2 (COL5A2) gene in family 4 (Figure 3D). This variant is also located in the COLFI domain, close to the C-terminal end (1,265–1,499 aa). The VarSite database also showed that a transition from a Gly to Glu side chain is substantial, and could likely lead to a modification in the protein’s function. Its occurrence in the gnomAD population database is exceedingly rare (PM2) and is likely to have a deleterious effect (PP3).

**FIGURE 4:**
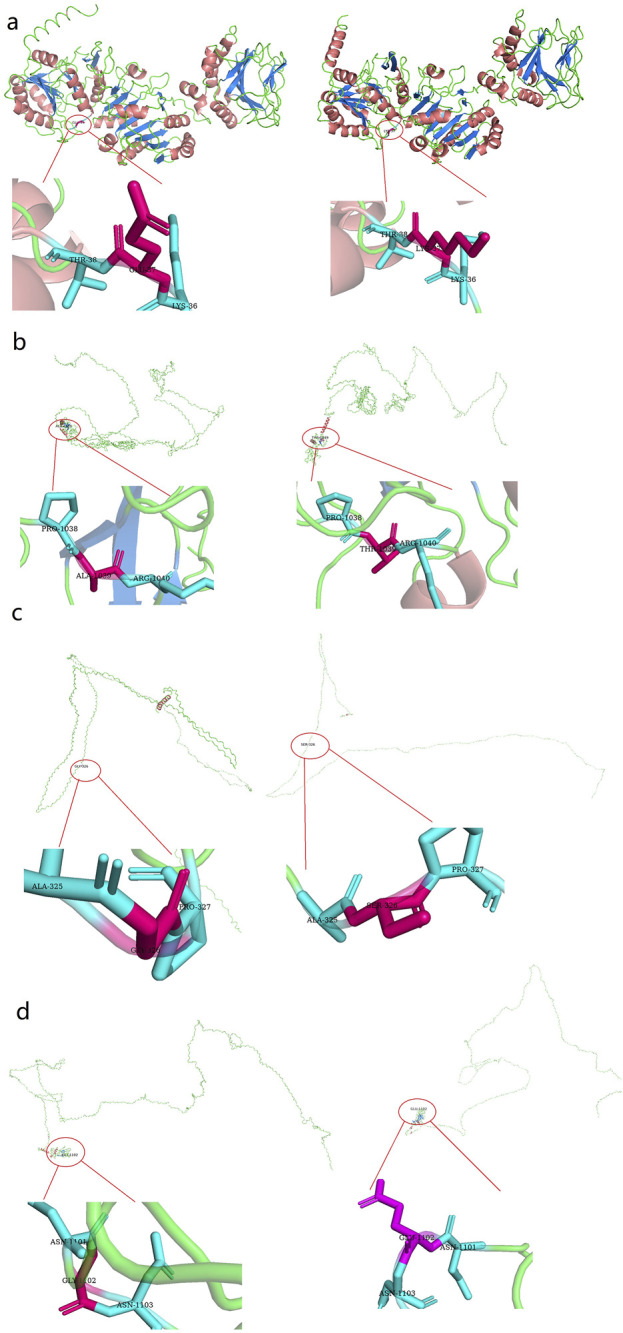
Three dimensional (3D) structures of the proteins show the sites of variants. The insert pictures are regional enlargements of the variants. **(a)** 3D modeling of wild-type *PLOD1* and p.Glu37Lys variant. **(b)** 3D modeling of wild-type *COL1A1* and p.Ala1256Ter variant. **(c)** 3D modeling of wild-type *COL4A1* and p.Glu326Ser variant; **(d)** 3D modeling of wild-type *COL5A2* and p.Gly1455Glu variant.

The above genotypes were co-segregated with the corresponding phenotypes. All of the missense variations were predicted to be damaging and highly conserved using software programs (Table 2). The conformational alteration of the proteins caused by these variations was revealed by 3D modeling and was compared with their wild-type proteins (Figures 4A–D). GERP++ scores indicated that these four variants were located in a highly conservative region. Based on the ACMG guidelines, these four variations were predicted to be variants of uncertain significance (VUS) (Table 2; Supplementary Table S1).

**TABLE 2:** Computational predictions and ACMG classification of all identified variants and their frequency in the gnomAD genomes.

Gene	Nucleotide	Amino acid	Polyphen2 prediction	GERP++	SIFT prediction	Provean	Mutation taster prediction	Fathmm-MKL	gnomAD_genomes	1 KG data [MAF (%)]
*PLOD1 (1p36.22)*	c.109G>A (NM_000302.4)	p.E37K	Possibly damaging	7.932	Tolerable	Deleterious	Disease causing	Deleterious	6.46E-05	0
*COL5A2 (2q32.2)*	c.4364G>A (NM_000393.5)	p.G1455E	Probably damaging	4.8	Deleterious	Deleterious	Disease causing	Deleterious	0	0
*COL1A1 (17q21.33)*	c.3766G>A (NM_000088.4)	p.A1256T	Probably damaging	3.93	Deleterious	Deleterious	Disease causing	Deleterious	6.47E-05	0.000998
*COL4A1 (13q34)*	c.976G>A(NM_001303110.2)	p.G326S	Probably damaging	4.79	Deleterious	Deleterious	Disease causing	Deleterious	0	0

Gene	Nucleotide	Amino acid	CADD score	Significance	ACMG	VarSite database
*PLOD1 (1p36.22)*	c.109G>A (NM_000302.4)	p.E37K	28.3	Likely deleterious	Uncertain significance (PM2_Supporting)	https://www.ebi.ac.uk/thornton-srv/databases/cgi-bin/VarSite/GetPage.pl?uniprot_acc=Q02809&template=resreport.html&res=E37K
*COL5A2 (2q32.2)*	c.4364G>A (NM_000393.5)	p.G1455E	26.7	Likely deleterious	Uncertain significance (PM2_Supporting+PP3)	https://www.ebi.ac.uk/thornton-srv/databases/cgi-bin/VarSite/GetPage.pl?uniprot_acc=P05997&var=G1455E
*COL1A1 (17q21.33)*	c.3766G>A (NM_000088.4)	p.A1256T	25.4	Likely deleterious	Uncertain significance (PM2_Supporting+PP3_Moderate+PS4_Moderate)	https://www.ebi.ac.uk/thornton-srv/databases/cgi-bin/VarSite/GetPage.pl?uniprot_acc=P02452&template=resreport.html&res=A1256T
*COL4A1 (13q34)*	c.976G>A(NM_001303110.2)	p.G326S	24.8	Possibly deleterious	Uncertain significance (PM2_Supporting+PP3_Moderate)	https://www.ebi.ac.uk/thornton-srv/databases/cgi-bin/VarSite/GetPage.pl?uniprot_acc=P02462&template=resreport.html&res=G326S

### Topological analyses of the PPI networks

**FIGURE 5:**
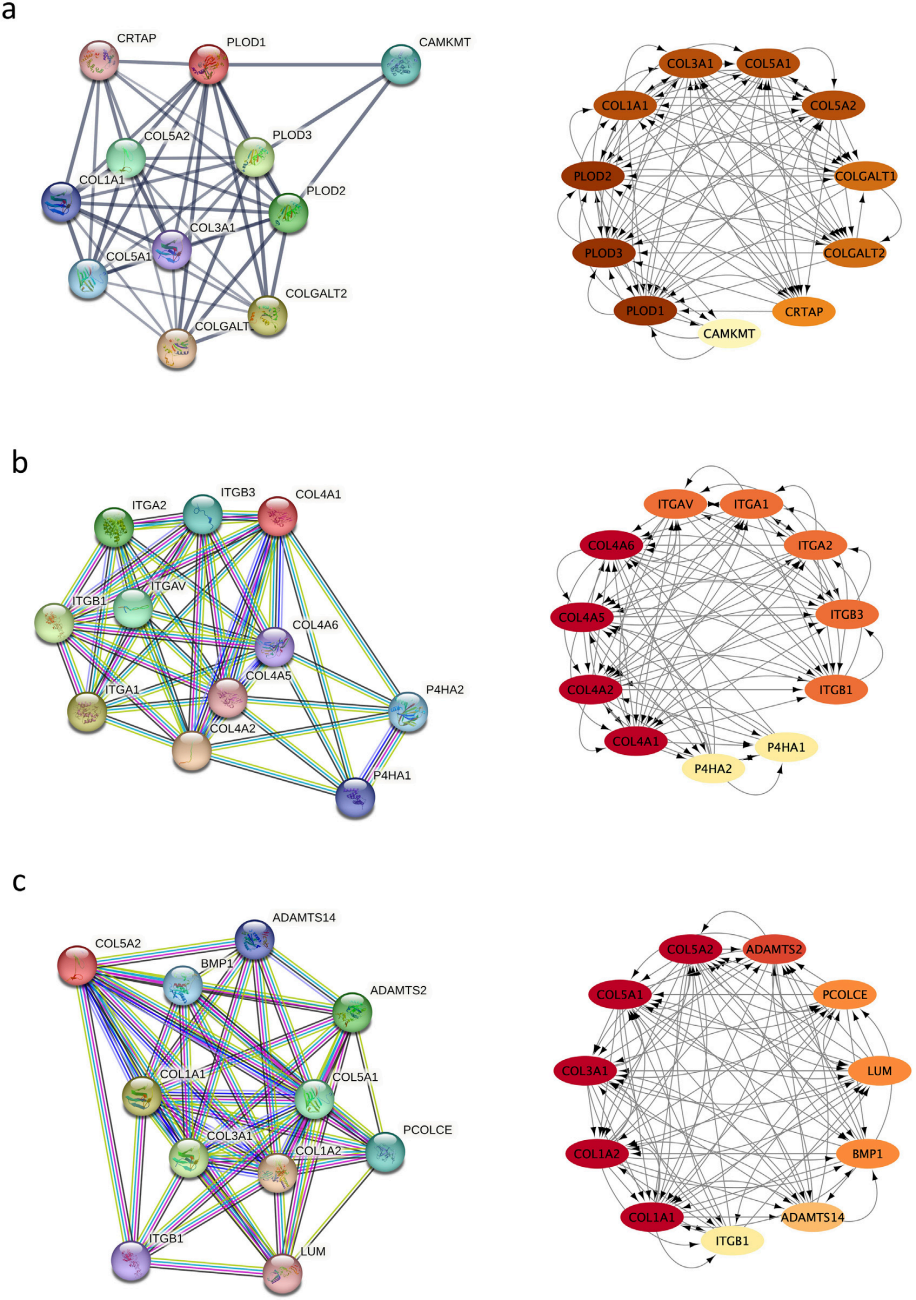
Protein–protein interaction (PPI) network. **(a)** the interaction of PLOD1 with COL1A1, COL4A1, and COL5A2; **(b)** the interaction of COL4A1 with ITGA and ITGB; **(c)** the interaction among COL1A1, COL1A2 and CO5A1, and their interaction with ITGB.

Using STRING, 11 significantly upregulated genes were updated to build the PPI networks of PLOD1, COL4A1, COL1A1, and COL5A2 (Figure 5A). Then the subnetworks were constructed using Cytoscape. In Figure 5B, the interaction of COL4A1 with integrin subunit alpha (ITGA) and ITGB are shown. Integrin is an important component of the extracellular matrix (ECM) involving in cytoskeleton remodeling, cell adhesion and migration, and integrin outside-in signaling, etc., which are involved in corneal development (https://www.wikipathways.org/pathways). In Figure 5C, the interaction among COL1A1, COL1A2 and COL5A1, and their interaction with ITGB are shown, which involves the assembly of collagen fibrils and other multimeric structures, integrin inside-out signaling, integrin-mediated cell adhesion and migration, ECM remodeling, and cytoskeleton remodeling, etc. (https://pubchem.ncbi.nlm.nih.gov/pathway;and https://www.genecards.org)

## Discussion

The etiology of KC is complex and involves environmental and genetic factors. Reduced corneal collagen or abnormal distribution of the collagen fibers are pathologic features of KC. In this study, four variants in *COL1A1*, *COL5A2*, *COL4A1*, and *PLOD1* genes were identified in four Chinese families with KC. *COL1A1*, *COL5A2*, and *COL4A1* are collagen genes and the *PLOD1* gene is associated with collagen crosslinking. The variants detected in the present study were all computationally predicted to be damaging and pathogenic for KC; however, further verification is warranted.

Before discussing the results of this study, a review of key activities related to corneal collagen and its crosslinkage should provide an understanding of the biomedical implication of the findings. In collagens, lysyl hydroxylase (LH) is important for catalyzing the hydroxylation of lysine residues in -X-LysGly- triplets (Kivirikko and Pihlajaniemi, 1998). LH has three isoenzymes: LH1, LH2, and LH3 (Ruotsalainen et al., 1999). The hydroxylysine residues are crucial for stability of the intermolecular crosslinks to ensure the mechanical stability and tensile strength of collagen fibrils, as well as the attachment sites for carbohydrate units (Gao et al., 2013). The formation of collagen crosslink takes place in the extracellular matrix and begins with the transformation of certain lysine or hydroxylysine residues in the telopeptides, catalyzed by lysyloxidase (Kagan and Li, 2003). All three primary fibril-forming collagens (types I, II, and III) possess four crosslinking sites, each in its own telopeptide and two within the triple helical region, near the N- and C-terminal ends (Eyre and Wu, 2005). Pyridinoline crosslinks are abundant in bone, tendons, ligaments, and cornea (Eyre and Wu, 2005). The hydroxylysine-linked carbohydrate units play a crucial role in controlling the formation and morphology of collagen fibrils(Notbohm et al., 1999) as well as type IV collagen-mediated crosslinking (Rautavuoma et al., 2004; Ruotsalainen et al., 2006). In Ehlers-Danlos syndrome (EDS), an inherited connective tissue disorder, LH1 deficiency caused by variants in the *PLOD1* gene is associated with the kyphoscoliotic subtype of EDS (Yeowell and Walker, 2000; Wilcox, 2003; Myllyharju and Kivirikko, 2004). In this subtype, ocular fragility cases and KC development have been reported (Wilcox, 2003; Myllyharju and Kivirikko, 2004).

**FIGURE 6:**
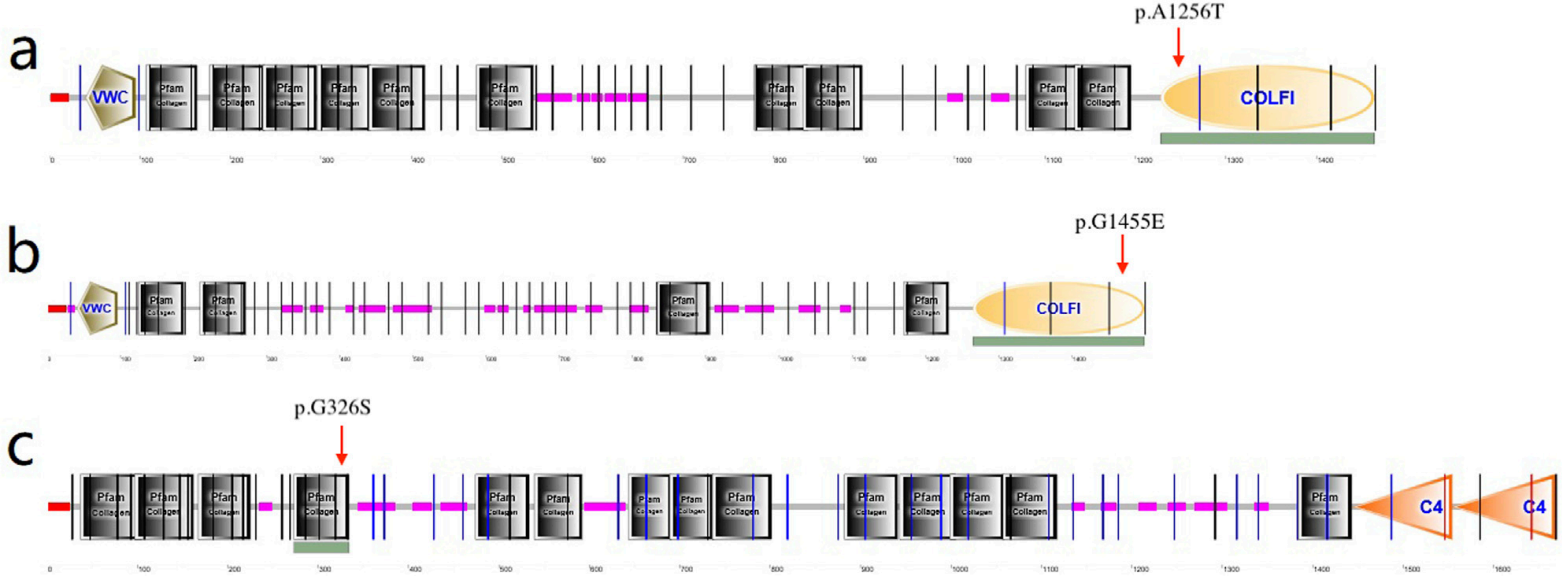
**(a)** p.Ala1256Ter amino acid change in COL1A1 located in the COLFI domain (arrow indicates 1,228–1,464aa); **(b)** p.Gly1455Glu in COL5A2 located in the COLFI domain (arrow indicates 1,265–1,499aa); **(c)** p.Gly326Ser in COL4A1 located in the Pfarm collagen domain (arrow indicates 273–334aa).

*COL1A1*, *COL5A2*, and *COL4A1* encode type I, IV, and V collagens, which are the primary constituents of the corneal stroma and basement membranes (Chan and Snibson, 2013). The composition of collagens in the stroma is 80%–90% type I and 10%–20% type V, whereas type IV collagen is one of the main components of basement membranes. The quantities of collagen type I and type V are vital for preserving the organization of the stroma as well as the shape and robustness of the cornea (Chakravarti et al., 1998; Quantock et al., 2003). Collagen type I forms collagen type V fibrils. Then the fibril growth and diameter are determined by the protrusion of nonhelical terminal extensions of collagen type V (Linsenmayer et al., 1993). Thus the variants in *COL1A1* and *COL5A2* could cause the abnormalities of collagen synthesis. In this research, a heterozygous variant c.3766G>A in *COL1A1* was identified in family 2 and it caused alteration in amino acid (p.Ala1256Thr), which is located in the fibrillar collagen C-terminal (COLFI) domain, 1,228–1,464 aa, one of the crosslinking sites (Figure 6A). Amino acid change in this region may cause protein dysfunction and deformation. Besides, in VarSite database, for wildtype, at sequence position 1,256 in this protein, the residue is an alanine which has an aliphatic side chain (i.e., containing only carbon and hydrogen atoms), which is hydrophobic (i.e., tending to avoid water and preferring to interact with other hydrophobic side chains), but the variant residue is a threonine which has a neutral side chain, this change may affect the structure stability.

In family 4, the variant in *COL5A2* (c.4364G>A, p.Gly1455Glu) is within the fourth cross-linking site (Figure 6B), and is expected to be highly conserved across species and causes potential harm. For wild-type, at sequence position 1,455 in this protein, the residue is a glycine which has no side chain, giving the protein increased flexibility at this location; the variant residue is a glutamic acid, possessing a negatively charged side chain, making it hydrophilic (i.e., favoring the protein’s surface over its interior).

Corneal basement membranes are essential structures in tissue development and maintenance. The main protein in the basement membranes, type IV collagen, is a family of six proteins encoded by six distinct genes: *COL4A1*, *COL4A2*, *COL4A3*, *COL4A4*, *COL4A5*, and *COL4A6* (Bai et al., 2009). *COL4A3*, *COL4A4*, and *COL4A5* have been identified as KC candidate genes (Bochert et al., 2003; Stabuc-Silih et al., 2009; Krolo et al., 2021). In 2020, Tian et al. (2020) investigated the role of the XIST-miR-181a-*COL4A1* axis in the development of KC and suggested that *COL4A1* might be correlated with KC. However, to date, *COL4A1* variants in KC families have not been reported. In this study, missense variation c.976G>A (p.Gly326Ser) was detected in the *COL4A1* gene and was predicted to be damaging. The substitution p.Gly326Ser was in the Pfam collagen domain (273–334 aa) (Figure 6C), which is predominantly repeats of the G-X-Y and polypeptide chain-formed triple helix and is considered highly conserved. This is the first time a *COL4A1* variation in a family with KC has been identified.

In this study, another variation detected was in the *PLOD1* gene. *PLOD1* is located on chromosome 1, which consists of 727 aa and is expressed in corneal stroma. *PLOD1* is expressed in corneal collagens and is associated with collagen crosslinking (Burdon et al., 2008). In 2017, Hudson et al. also confirmed a common lysine under-hydroxylation effect at the helical domain crosslinking sites in the skin, bone, tendon, aorta, scleral, and cornea (Hudson et al., 2017). In our study, a heterozygous *PLOD1* mutation c.109G>A was identified in family 1. The structural change in the protein induced by this variant may disturb the crosslinking of corneal fibrils. In addition, PPI network showed the interaction between PLOD1 and COL1A1 or PLOD1 and COL5A1. Therefore, misfolding and/or aggregate formation of PLOD1 likely cause the development of KC by disturbing corneal crosslinking. To date, this is the first report to identify a *PLOD1* variation in a family with KC.

Clinically, the corneal crosslinking (CXL) technique uses riboflavin and UVA light to treat KC. The mechanism of this treatment is that UVA, in conjunction with riboflavin, initiates oxidative and glycosylation processes, resulting in the generation of collagen crosslinks and strengthening the corneal stroma (Gomes et al., 2015; Jacob et al., 2018). However, corneal structures such as endothelial cells can potentially be damaged after CXL, leading to corneal edema and vision loss (Bagga et al., 2012; Sharma et al., 2012). Moreover, transient or persistent corneal haze after CXL and post-CXL infectious keratitis have been reported (Kim et al., 2016; Kodavoor et al., 2020). CXL also cannot be performed in patients who are pregnant or breastfeeding or have eye inflammation, pronounced corneal scarring, autoimmune diseases, or corneal thickness <400 µm. In view of the limitations of CXL surgery and the risk of complications, treatment with collagen crosslinking without these issues should be developed. Since the *PLOD1* gene plays an important role in regulating collagen crosslinking, gene therapy for the variations in *PLOD1* could be a treatment strategy to be considered.

## Conclusion

In conclusion, through investigating four Chinese families with KC, four variants in *COL1A1*, *COL5A2*, *COL4A1*, and *PLOD1* genes were identified in this study. *COL1A1*, *COL5A2*, and *COL4A1* are collagen genes and *PLOD1* is a collagen crosslinking regulatory gene. These discoveries broaden the range of recognized gene variants in KC and warrant additional studies on these variants.

## Data Availability

The datasets presented in this study can be found in online repositories. The names of the repository/repositories and accession number(s) can be found below: https://www.ncbi.nlm.nih.gov/, SCV004030248, SCV004030250, SCV004030251 and SCV004030252.

## Supplementary material

SUPPLEMENTARY FIGURE S1 Conservation analysis diagram of the variants. **(a)** Conservation analysis of the amino acid position where the p.Glu37Lys of the *PLOD1* gene is located among various species; **(b)** Conservation analysis of the amino acid position where p.Ala1256Thr of the *COL1A1* gene is located among various species; **(c)** Conservation analysis of the amino acid position where p.Glu326Ser of the *COL4A1* gene is located among various species; **(d)** Conservation analysis of the amino acid position where p.Gly1455Glu of the *COL5A2* gene is located among various species.

SUPPLEMENTARY TABLE S1 Variants evaluated in the four families with KC.

## Abbreviations

KC, Keratoconus; UVA, ultraviolet-A; UCVA, uncorrected visual acuity; BSCVA, best spectacle corrected visual acuity; CC, corneal curvature; CTTP, corneal thickness at the thinnest point; ES, exome sequencing; SIFT, Solar Instant Feasibility Tool; PCR, polymerase chain reaction; 3D, three-dimensional; PPI, protein-protein interaction; PEC, posterior elevation of the cornea; MASCs, maximum anterior surface curvatures; CCTs, central corneal thicknesses; *PLOD1*, procollagen-lysine, 2-oxoglutarate 5-dioxygenase 1; *COL1A1*, collagen type I alpha 1; *COL4A1*, collagen type IV alpha 1; *COL5A2*, collagen type V alpha 2; LH, lysyl hydroxylase; EDS, Ehlers-Danlos syndrome; COLFI, fibrillar collagen C-terminal; CXL, corneal crosslinking.
