# Supplementary figures and images for: Novel variations in the PLOD1, COL1A1, COL5A2 and COL4A1 genes related to keratoconus
Source: Front Genet. 2025 Mar 25;16:1497915. doi: 10.3389/fgene.2025.1497915 (PMC11975878; doi:10.3389/fgene.2025.1497915)

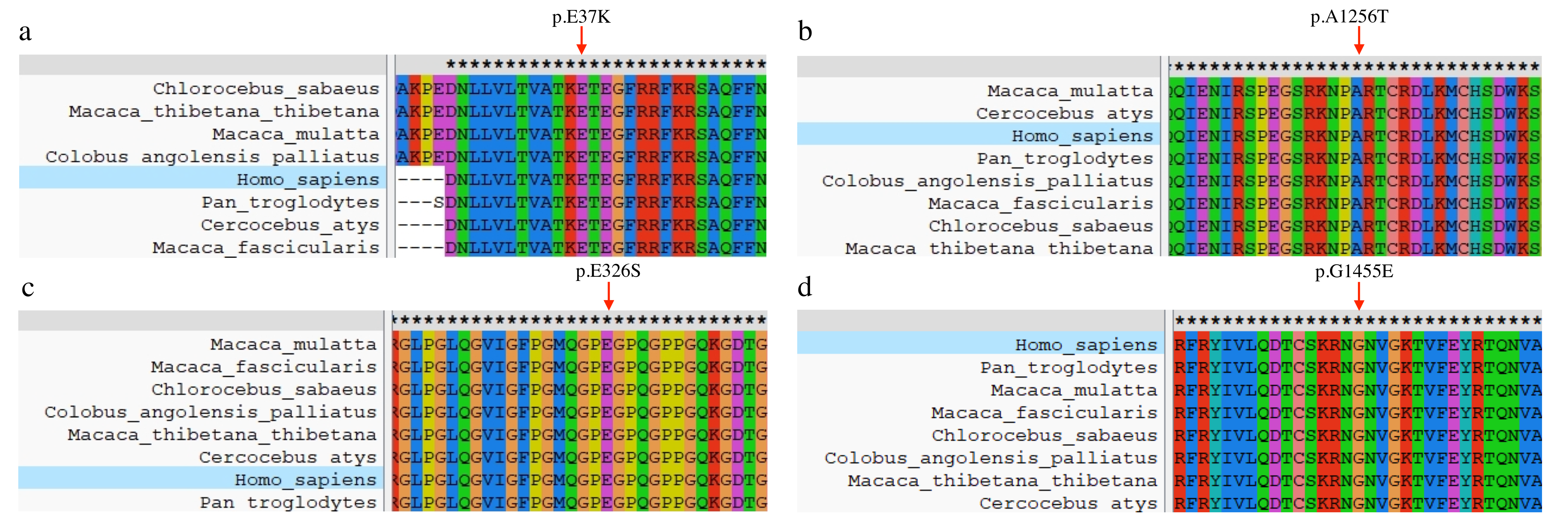

Supplement: Supplementary file 1 [file Image1.tif]
